# MOTHER-CHILD RELATIONSHIP IN THE CONTEXT OF DOMESTIC VIOLENCE

**DOI:** 10.1192/j.eurpsy.2023.2391

**Published:** 2023-07-19

**Authors:** A. Boukdir

**Affiliations:** 1 Psychiatry, University Psychiatric Hospital Ar-razi of Salé, University Hospital Centre Ibn Sina, Temara, Morocco

## Abstract

**Introduction:**

Mother-child relationship has a major role in a child’s cognitive, emotional and behavior shaping. Unfortunately, in the context of domestic violence, this relationship can be negatively impacted becoming strained or distant.

**Objectives:**

To assess the quality of Mother-Child Relationship in the context of domestic violence or intimate partner violence. And to investigate the factors influencing negatively the Mother-Child Relationship.

**Methods:**

This is a descriptive and analytical cross-sectional study, conducted among abused women and their children, recruited from associations combatting violence against women, from Moulay abdellah hospital of Salé, and from consultation at the university psychiatric hospital Arrazi of Salé, through a hetero-questionnaire that includes socio-demographic characteristics and scales measuring the quality of mother-child relationship (IPA, CAM) and psychological distress of the mother and the child (EMMDP).

**Results:**

From the results observed in women and children recruited in our study, we retain that various elements are impacting the mother-child relationship, such as psychological maternal functioning, child’s behavior functioning, parenting qualities, insecurity of child’s attachment, unhealthy internalized representations…

**Conclusions:**

A better understanding of the factors influencing mother-child relationship, can allow us to offer more tailored supports and could enable interventions promoting recovery and women’s and children’s well-being in the context of domestic violence.

**Disclosure of Interest:**

None Declared

